# Rho Kinase in Eye Disease

**DOI:** 10.1155/2017/9281745

**Published:** 2017-08-27

**Authors:** Naoki Okumura, Shintaro Nakao, Toshihiro Inoue, Padmanabhan Pattabiraman

**Affiliations:** ^1^Department of Biomedical Engineering, Faculty of Life and Medical Sciences, Doshisha University, Kyotanabe, Japan; ^2^Department of Ophthalmology, Graduate School of Medical Sciences, Kyushu University, Fukuoka, Japan; ^3^Department of Ophthalmology, Kumamoto University, Kumamoto, Japan; ^4^Department of Ophthalmology, Case Western Reserve University, Cleveland, OH, USA

Rho-associated protein kinase (ROCK) is a well-characterized effector of Rho GTPase, a small GTP-binding protein. The Rho/ROCK signaling pathways contribute to a wide range of fundamental cellular events, such as cell adhesion, motility, proliferation, differentiation, and apoptosis. The role of ROCK in the control of a wide spectrum of biological events has made it a subject of intensive investigation as an important therapeutic target in a wide range of diseases, including vascular disease, cancer, neuronal degenerative disease, asthma, and glaucoma.

The ROCK inhibitor, fasudil, was approved in 1995 for the prevention of cerebral vasospasm in patients with subarachnoid hemorrhage. In 2014, another ROCK inhibitor, ripasudil, was approved in Japan for the treatment of glaucoma and ocular hypertension. More importantly, the research supporting the involvement of ROCK signaling in glaucoma has now extended to other eye diseases, such as corneal endothelial disease, cataract, age-related macular degeneration, and proliferative vitreous retinopathy.

This special issue focuses on the involvement of ROCK signaling in the pathobiology of eye-related diseases and the possible applications of ROCK inhibitors as drugs for treating these disorders.

Numerous researchers have devoted their efforts toward the development of ROCK inhibitors that can target glaucoma. ROCK inhibitors alter the distribution of actin stress fibers and modulate the cell-matrix interactions of cultured trabecular meshwork and Schlemm's canal cells. In addition, topical administration of ROCK inhibitor eye drops reduces the intraocular pressure (IOP) in rabbit and monkey models by increasing the outflow capacity. Several ROCK inhibitors also reduced IOP in healthy human volunteers, as well as in patients with glaucoma and ocular hypertension in clinical studies. As mentioned above, ripasudil was approved in Japan as a first-in-class ROCK inhibitor ophthalmic agent for the treatment of glaucoma and ocular hypertension. Y. Kaneko et al. showed that a specific ROCK inhibitor, ripasudil hydrochloride hydrate, had an additional IOP-lowering effect in rabbit or monkey models when used in combined regimens that included *β*-blockers, *αβ*-blockers, *α*2-agonists, carbonic anhydrase inhibitors, and prostaglandin analogs. Their study indicated that ripasudil has a particular efficacy when used in combination with conventional eye drops. Ripasudil is a first-in-class ROCK inhibitor eye drop, so further basic and clinical data regarding its safety and effectiveness will be beneficial, especially for clinicians.

The use of ROCK inhibitors has been proposed for the treatment of corneal endothelial decompensation. N. Okumura et al. reviewed the usefulness of ROCK inhibitors both as eye drops and as adjunct drugs in cell-based therapies. They showed that ROCK inhibitors, supplied in the form of eye drops, promoted cell proliferation in animal models, and pilot clinical research suggests the occurrence of a similar response in humans as well. They have predicted the future use of topically applied ROCK inhibitors for the treatment of (1) Fuchs endothelial corneal dystrophy, combined with central corneal endothelial removal, and (2) acute corneal endothelial damage induced by cataract surgery. Along these lines, A. Akhbanbetova et al. designed a cryoprobe for corneal endothelial removal prior to ROCK inhibitor administration as part of a treatment for Fuchs endothelial corneal dystrophy. They demonstrated that their cryoprobe destroys the corneal endothelium in a consistent manner, without altering the collagen fibril structure of the corneal stroma. N. Okumura et al. have also reviewed the accumulating evidence supporting the usefulness of ROCK inhibitors as adjunct drugs in a cell-based therapy. The first-in-man clinical trial of the cell-based therapy combined with a ROCK inhibitor as an adjunct drug for the treatment of corneal endothelial dysfunction was initiated in Japan in 2013. Future clinical assessments are necessary, but the potential for ROCK inhibitors to target the corneal endothelium is very promising.

ROCK inhibitors have also been researched as potent pharmaceutical agents for targeting retinal diseases, such as wet age-related macular degeneration, diabetic retinopathy, diabetic macular edema, and proliferative vitreoretinopathy. In addition to the preclinical data obtained using animal models, several clinical studies have now demonstrated a positive effect of ROCK inhibitors on retinal diseases. M. Yamaguchi et al. reviewed the recent progress on the mechanisms of ROCK signaling in vitreoretinal diseases and the potency of their clinical application. Vitreoretinal diseases are strongly modulated by vascular endothelial growth factor (VEGF), so intravitreous injection of anti-VEGF agents has been extensively used in the clinical setting. M. Yamaguchi et al. have reviewed the potent effects of ROCK inhibitor administration on each biological process involved in eye disease and have carefully compared ROCK inhibitor effects with those of anti-VEGF agents.

J. S. Moon et al. demonstrated in a rabbit model that intraoperative subconjunctival injection of ROCK inhibitor suppressed the inflammation and fibrosis that typically occur after extraocular muscle surgery. ROCK inhibitors are known to trigger an antifibrotic effect in several cell types in the eye, such as Tenon fibroblasts, the trabecular meshwork, retinal pigment epithelial cells, hyalocytes, and corneal stroma cells. Human clinical trials have not yet recapitulated this antifibrotic effect of ROCK inhibitors, but this phenomenon is worth further investigation.

Thus, growing evidence supports the usefulness of ROCK inhibitors as promising therapeutic modalities for the treatment of various ophthalmological disorders. We expect that this special issue will provide a cross-sectional platform for readers to discover the importance and possible future applications of ROCK inhibitors in the treatment of eye diseases.

*Naoki Okumura* *Shintaro Nakao* *Toshihiro Inoue* *Padmanabhan Pattabiraman*

